# Heart Rate Variability Frequency-Domain Analysis Across Glaucoma Subtypes

**DOI:** 10.3390/biomedicines13081805

**Published:** 2025-07-23

**Authors:** Misaki Ukisu, Yuto Yoshida, Hinako Takei, Keigo Takagi, Masaki Tanito

**Affiliations:** Department of Ophthalmology, Shimane University Faculty of Medicine, Izumo 693-8501, Japany-yoshida@juntendo.ac.jp (Y.Y.);

**Keywords:** frequency-domain heart rate variability, autonomic nervous system, primary open-angle glaucoma, exfoliation glaucoma

## Abstract

**Background/Objectives**: Heart rate variability (HRV) is a marker of autonomic nervous system function, based on fluctuations in heartbeat intervals. Although several studies have investigated the association between frequency-domain HRV parameters and glaucoma, evidence based on large sample sizes remains limited. Therefore, the present study aimed to examine the relationship between frequency-domain HRV parameters and glaucoma subtypes, including primary open-angle glaucoma (PG) and exfoliation glaucoma (EG), using a larger sample size. **Methods**: Participants with primary open-angle glaucoma (PG), exfoliation glaucoma (EG), or no ocular disease other than cataract (controls) were recruited at Shimane University between June 2023 and July 2024. Frequency-domain HRV parameters (total power [TP], very-low-frequency [VLF], low-frequency [LF], high-frequency [HF], and LF/HF) were measured using a sphygmograph (TAS9 Pulse Analyzer Plus View). Group comparisons were conducted using unpaired *t*-tests, Fisher’s exact tests, and Tukey’s HSD test. Multivariate analyses were performed to identify factors associated with each HRV parameter. **Results**: A total of 809 participants were analyzed, including 522 with PG, 191 with EG, and 96 controls. The EG group showed significantly lower values across all frequency-domain HRV parameters compared to the PG group, and significantly lower LnLF values than the control group (*p* = 0.012). Multivariate analyses revealed that no significant associations were found between HRV measures and the presence of glaucoma or pseudoexfoliation material (PEM) deposition. Older age was significantly associated with lower values across all HRV parameters. **Conclusions**: In elderly glaucoma patients, age-related alterations in frequency-domain HRV parameters have been observed.

## 1. Introduction

The autonomic nervous system (ANS) exerts regulatory control across most organ systems in the human body. Structurally, it is divided into three major components: the sympathetic, parasympathetic, and enteric systems [1]. ANS dysfunction may influence the onset and progression of various diseases, including psychiatric disorders [2], neurodegenerative diseases [3,4], metabolic diseases [5,6], and cardiovascular diseases [7,8].

ANS dysfunction may also be associated with glaucoma [9,10,11,12,13]. Heart rate variability (HRV), which reflects fluctuations in the time intervals between successive heartbeats and serves as an indicator of autonomic balance, has been widely used to assess ANS function [14]. Asefa et al. reported that lower HRV was associated with a higher prevalence of glaucoma, with individuals in the lowest HRV quartile showing a 15% increased risk compared to those in the highest quartile [15]. A previous study has shown that patients with normal-tension glaucoma (NTG) exhibit significantly reduced HRV during both daytime and nighttime periods [16]. According to Liu et al., decreased HRV may be a predictor of more rapid deterioration in glaucoma patients [10]. Therefore, detecting changes in the ANS may be useful for understanding the pathophysiology of glaucoma and for making clinical prognostic assessments.

However, there are still few studies with large sample sizes that have examined the association between glaucoma and autonomic dysfunction. HRV includes time-domain analysis, which statistically processes raw time-series data, and frequency-domain analysis, which allows for estimation of autonomic nervous system components by analyzing the frequency spectrum. Our group previously investigated the association between time-domain parameters of HRV and glaucoma subtypes using a large sample size, and found that patients with exfoliation glaucoma (EG) exhibited significantly lower standard deviation of normal-to-normal intervals (SDNN) and coefficient of variation of R–R intervals (CVRR) than those with primary open-angle glaucoma (PG) [17]. Nevertheless, the association between frequency-domain HRV parameters and glaucoma remains unclear. To address these gaps in the current literature, the present study explores the relationship between frequency-domain HRV parameters and glaucoma subtypes, including PG and EG, based on a larger sample size. As a result, it was found that changes in the ANS were more pronounced in EG, which predominantly affects older individuals.

## 2. Materials and Methods

### 2.1. Study Design and Participants

This cross-sectional study was approved by the Institutional Review Board of Shimane University Hospital (approval number: 20200228-2; revised on 27 October 2024) and conducted in accordance with the principles of the Declaration of Helsinki. The objective was to investigate the association between ANS activity, as assessed by frequency domain HRV, and different glaucoma subtypes. Participants were recruited at Shimane University between June 2023 and July 2024. The study included Japanese individuals diagnosed with PG or EG, as well as control participants with no ocular conditions other than cataract. Instead of obtaining written informed consent, information regarding the study was published on the institutional website, allowing participants the opportunity to opt out. Exclusion criteria were as follows: (1) an HRV reliability score below 95%, and (2) the presence of ocular conditions other than PG, EG, or cataract. One eye per participant was selected for analysis. For control subjects, the eye with better visual acuity was chosen; if both eyes had equal acuity, the right eye was used. In cases of unilateral PG or EG, the affected eye was selected. For patients with bilateral glaucoma, the eye with the higher intraocular pressure (IOP) was chosen; if IOP was equal in both eyes, the right eye was analyzed. The diagnosis of glaucoma was confirmed by ophthalmologists based on comprehensive ocular examinations, including IOP measurement, slit-lamp examinations, gonioscopy, optic nerve evaluation using fundus photography and optical coherence tomography (OCT), and visual field testing. The diagnosis of PG was made based on the presence of open iridocorneal angles in both eyes, glaucomatous optic nerve damage characterized by enlarged optic disc cupping or localized thinning of the neuroretinal rim, and matching visual field defects in at least one eye, with no evidence of secondary glaucoma in either eye. EG was identified by the presence of pseudoexfoliation material on the lens capsule and/or pupillary margin in one or both eyes, along with an open iridocorneal angle. Visual field abnormalities were evaluated using an automated perimeter (Humphrey Visual Field Analyzer, Carl Zeiss Meditec, Dublin, CA, USA).

### 2.2. Frequency Domain Heart Rate Variability

Frequency domain HRV analysis was performed to evaluate ANS activity based on the distribution of power across specific frequency bands. Measurements were conducted using a sphygmograph device (TAS9 Pulse Analyzer Plus View; YKC Corp., Tokyo, Japan) configured for frequency-domain analysis. All recordings were obtained with participants seated in a resting state during daytime outpatient visits (measurements were conducted in the morning for most patients), using a standardized 20 s measurement protocol with a sampling frequency of 1 kHz. All measurements were performed by ophthalmic technicians experienced in this examination. Each patient underwent the test once; however, if the technician judged that the result was not stable, the measurement was repeated.

In this study, the following frequency-domain HRV parameters [18,19,20] were used: total power (TP), which reflects the overall variance in heart rate and total autonomic activity; very-low-frequency power (VLF; 0.0033–0.04 Hz), associated with thermoregulation and other long-term regulatory mechanisms; low-frequency power (LF; 0.04–0.15 Hz), which reflects both sympathetic and parasympathetic activity and is influenced by baroreflex function; high-frequency power (HF; 0.15–0.40 Hz), primarily indicative of parasympathetic activity; and the LF/HF ratio, often interpreted as an index of sympathovagal balance, with higher values suggesting sympathetic dominance. To normalize the distributions and facilitate statistical analysis, each parameter was transformed using the natural logarithm, resulting in LnTP, LnVLF, LnLF, LnHF, and LnLF/HF, which were used in subsequent analyses.

### 2.3. Statistical Analysis

Descriptive statistics are presented as means ± standard deviations (SD) for continuous variables and as percentages for categorical variables. Comparisons of demographic characteristics and HRV parameters between the control and glaucoma groups were performed using unpaired *t*-tests for continuous variables and Fisher’s exact tests for categorical variables. For pairwise comparisons among the control, PG, and EG groups, post hoc analyses were conducted using Tukey’s honest significant difference (HSD) test. Multivariate analyses were further performed to examine potential factors associated with each frequency-domain HRV parameter (LnTP, LnVLF, LnLF, LnHF, and LnLF/HF). These analyses employed generalized linear regression models, with the following covariates included as potential confounders: age, sex, smoking status, body mass index (BMI), systolic blood pressure (sBP), diastolic blood pressure (dBP), pulse rate, hypertension, diabetes mellitus (DM), and presence of pseudoexfoliation material (PEM). A *p*-value < 0.05 was considered statistically significant. All statistical analyses were performed using JMP Pro version 17.2.0 (SAS Institute Inc., Cary, NC, USA).

## 3. Results

A total of 809 participants (one eye per participant) were included in the analysis. Participant characteristics are summarized in Table 1. Among them, 713 were diagnosed with glaucoma: 522 had PG, and 191 had EG. The remaining 96 participants without ocular disease other than cataract were assigned to the control group. The mean age ± SD was 68.6 ± 12.5 years for all glaucoma patients, with subgroup means of 66.0 ± 12.7 years in the PG group, 75.8 ± 8.7 years in the EG group, and 59.9 ± 18.8 years in the control group. Statistically significant differences in age were observed between the control and glaucoma groups, as well as among all subgroup comparisons (control vs. PG, control vs. EG, and PG vs. EG; all *p* < 0.0001). In addition to age, statistically significant differences were observed between the control and glaucoma groups in pulse rate, hypertension status, and the presence of PEM. The mean pulse rate was higher in the control group compared to the glaucoma group (80.1 ± 15.7 bpm vs. 73.6 ± 12.6 bpm, *p* < 0.0001). The prevalence of hypertension was also significantly lower in the control group (29.2%) than in the glaucoma group (45.0%, *p* = 0.003). Furthermore, while no PEM deposition was observed in the control group, it was present in 26.8% of glaucoma patients (*p* < 0.0001).

Table 2 summarizes the comparison of frequency domain HRV parameters across the control, glaucoma, PG, and EG groups. The mean LnTP was significantly different among the groups (*p* = 0.03), with a notable difference between the PG and EG groups (*p* = 0.03). For LnVLF, a significant overall difference was found (*p* = 0.02), primarily driven by a significant difference between the PG and EG groups (*p* = 0.02). LnLF showed significant group differences (*p* = 0.002), with post hoc tests revealing lower values in the EG group compared to both the control group (*p* = 0.01) and the PG group (*p* = 0.04). LnHF also demonstrated a significant overall difference (*p* = 0.02), although no significant differences were identified in post hoc pairwise comparisons. Regarding the sympathovagal balance, assessed by the LnLF/LnHF ratio, a significant group difference was observed (*p* = 0.01), with the EG group showing a significantly lower ratio compared to the PG group (*p* = 0.008).

As shown in Table 3, Table 4, Table 5, Table 6 and Table 7, multivariate regression analyses for the frequency-domain HRV parameters (LnTP, LnVLF, LnLF, LnHF, and LnLF/LnHF) identified several significant associations. Older age was significantly associated with lower values across all HRV parameters. Likewise, a higher pulse rate was consistently associated with reduced HRV in all models. Hypertension was negatively associated with LnTP, LnLF, LnHF, and LnLF/LnHF. In addition, a higher BMI was significantly associated with lower LnTP, LnLF, and LnHF values. In contrast, neither PEM deposition nor the presence of glaucoma showed a significant association with any of the frequency domain HRV parameters in the multivariate models.

## 4. Discussion

This study represents a large-scale cross-sectional investigation of frequency-domain HRV across glaucoma subtypes, including PG and EG. As shown in Table 2, significant differences in HRV parameters were observed among the groups. Notably, the EG group demonstrated significantly lower values across all frequency-domain HRV parameters compared to the PG group. Additionally, the EG group exhibited significantly lower LnLF values than the control group. In multivariate analyses (Table 3, Table 4, Table 5, Table 6 and Table 7), older age, higher BMI, elevated pulse rate, and the presence of hypertension were all independently associated with decreased frequency-domain HRV parameters. In contrast, no significant associations were found between HRV measures and either the presence of glaucoma or PEM deposition. 

The association between frequency-domain HRV parameters and glaucoma remains unclear, with existing studies yielding inconsistent findings. Some studies have found elevated LF, HF, and LF/HF ratios in glaucoma patients, suggesting increased sympathetic activity [13,21], while others have observed decreases in these indices [11,12], particularly in NTG or high tension glaucoma (HTG) subtypes. However, the number of studies investigating frequency-domain HRV in glaucoma patients is limited. To our knowledge, the present study is the first to explore the link between frequency-domain HRV parameters and glaucoma using a larger sample size. The association between glaucoma and ANS dysfunction may be explained by several underlying mechanisms. One hypothesis involves impaired vascular regulation due to autonomic imbalance, particularly reduced parasympathetic and/or increased sympathetic activity, which could lead to unstable ocular perfusion and contribute to optic nerve damage [22,23]. In addition, autonomic dysfunction may alter IOP regulation by influencing aqueous humor production or outflow through neurohumoral pathways [24]. Systemic autonomic dysregulation has also been linked to oxidative stress and endothelial dysfunction, both of which are implicated in glaucoma pathogenesis [25,26]. These pathways suggest that ANS alterations may play a contributory role in both the development and progression of glaucoma.

In line with our previous study examining time-domain HRV in glaucoma [17], the current analysis revealed significantly lower frequency-domain HRV parameters in glaucoma patients, especially within the EG group. One possible explanation involves the underlying pathophysiology of exfoliation syndrome (XFS), which leads to EG. XFS has been associated with vascular abnormalities, such as impaired endothelial function [27], increased oxidative stress both ocularly and systemically [28,29], and abnormalities in coagulation processes [30]. These vascular changes may indirectly contribute to dysregulation of the autonomic nervous system. Further studies are warranted to clarify the mechanistic pathways linking EG and autonomic dysfunction.

In the univariate analysis, the EG group showed significantly reduced values in all frequency-domain HRV parameters compared to the PG group, and also exhibited markedly lower LnLF values relative to the control group. As this study included only glaucoma outpatients, the number of controls was relatively small compared to the PG and EG groups, which may have affected the results of the multivariate analysis. Furthermore, after adjusting for age, the differences in HRV parameters among groups were attenuated, possibly reflecting the greater prevalence of EG in older populations. In multivariate analysis, factors such as the presence of hypertension, blood pressure, pulse rate, and BMI were identified as influencing HRV parameters. It is well known that glaucoma is modulated not only by local ocular factors but also by various systemic conditions [31]. The disappearance of the association between glaucoma and HRV in the multivariate analysis may paradoxically suggest that complex systemic factors are deeply involved in the pathophysiology of glaucoma and EG. The results of the multivariate analysis indicate that the impact of HRV on glaucoma is not direct, but is largely mediated by systemic factors such as aging. However, the present findings suggest that in glaucoma subtypes that develop in older individuals, such as EG, changes in the ANS are more prominently detected compared to younger patients. Future studies with larger and more balanced cohorts are needed to confirm these findings and to further elucidate the relationship between glaucoma or EG and ANS regulation.

This study has several limitations. First, the potential for selection bias should be considered. As all participants were recruited from patients attending the glaucoma clinic at Shimane University Hospital, the control group may not be representative of the general healthy population. Second, the time of day at which HRV was measured was not taken into account. Given that HRV can vary depending on the time of measurement, this may have influenced the results. Third, the potential effects of anti-glaucoma and antihypertensive medications were not evaluated. Since these agents may alter IOP, BP, pulse rate, and HRV parameters, their exclusion may have introduced confounding effects. Fourth, due to the cross-sectional design, it is not possible to establish a causal relationship between frequency-domain HRV measures and glaucoma. We conducted analyses on the association between subject-based background factors and HRV parameters in the present study. In the future, we plan to investigate the relationship between eye-based background factors—such as intraocular pressure and visual field sensitivity—and HRV.

## 5. Conclusions

This is the first study to comprehensively evaluate frequency-domain HRV parameters across glaucoma subtypes, including PG and EG, in a large clinical sample. Patients with EG showed significantly lower HRV values compared to those with PG and healthy controls in unadjusted analyses. However, multivariate models revealed that frequency-domain HRV parameters were not associated with the presence of glaucoma or PEM deposition. These findings indicate that in elderly patients with glaucoma, particularly those with EG, age-related changes in ANS balance are present. Further longitudinal studies are needed to clarify causal relationships and underlying mechanisms.

## Figures and Tables

**Table 1:** Comparison of demographic data between control and glaucoma groups, and control group and PG group and EG group.

Parameters	Control		Glaucoma		PG		EG		*p* Value ^a^
	N or Mean ± SD	% or 95% CI Range	N or Mean ± SD	% or 95% CI Range	N or Mean ± SD	% or 95% CI Range	N or Mean ± SD	% or 95% CI Range	
Subjects	96		713		522		191		
Eyes	96		713		522		191		
Age, years	59.9 ± 18.8	56.0, 63.7	68.6 ± 12.5	67.7, 69.5	66.0 ± 12.7	64.9, 67.1	75.8 ± 8.7	74.5, 77.0	<0.0001 **
*p* value, vs. control ^b^			<0.0001 **		<0.0001 **		<0.0001 **		
*p* value, vs. PG ^b^							<0.0001 **		
Sex									
Male	55	57.3	389	54.6	285	54.6	104	54.5	0.89
Female	41	42.7	324	45.4	237	45.4	87	45.6	
*p* value, vs. control ^b^			0.66		0.66		0.71		
*p* value, vs. PG ^b^							>0.99		
Smoking habit									
yes	77	83.7	606	88.7	441	88.7	165	88.7	0.36
no	15	16.3	77	11.3	56	11.3	21	11.3	
*p* value, vs. control ^b^			0.17		0.17		0.26		
*p* value, vs. PG ^b^							>0.99		
BMI, kg/m^2^	23.2 ± 4.35	22.3, 24.1	22.7 ± 3.26	22.5, 23.0	22.7 ± 3.30	22.4, 23.0	22.8 ± 3.18	22.3, 23.2	0.52
*p* value, vs. control ^b^			0.26		0.49		0.64		
*p* value, vs. PG ^b^							0.52		
sBP, mmHg	141 ± 23.4	136, 146	143 ± 21.0	141, 144	141 ± 20.8	139, 143	148 ± 20.2	145, 151	0.0002 **
*p* value, vs. control ^b^			0.51		0.98		0.03 *		
*p* value, vs. PG ^b^							0.0001 **		
dBP, mmHg	79.5 ± 14.3	76.5, 82.5	80.7 ± 13.3	79.7, 81.7	80.8 ± 13.1	79.7, 82.0	80.3 ± 14.1	78.2, 82.3	0.64
*p* value, vs. control ^b^			0.42		0.65		0.88		
*p* value, vs. PG ^b^							0.88		
Pulse rate, bpm	80.1 ± 15.7	76.8, 83.4	73.6 ± 12.6	72.6, 74.6	73.0 ± 12.2	71.9, 74.1	75.3 ± 13.6	73.3, 77.3	<0.0001 **
*p* value, vs. control ^b^			<0.0001 **		<0.0001 **		0.01 *		
*p* value, vs. PG ^b^							0.10		
Hypertension									
yes	28	29.2	321	45.0	225	43.1	96	50.3	0.0029 **
no	68	70.8	392	55.0	297	56.9	95	49.7	
*p* value, vs. control ^b^			0.003 **		0.01 *		0.0007 **		
*p* value, vs. PG ^b^							0.09		
DM									
yes	20	20.8	100	14	69	13.2	31	16.2	0.12
no	76	79.2	613	86	453	86.8	160	83.8	
*p* value, vs. control ^b^			0.09		0.06		0.33		
*p* value, vs. PG ^b^							0.33		
PEM deposition									
yes	0	0	191	26.8	0	0	191	100	<0.0001 **
no	96	100	522	73.2	522	100	0	0	
*p* value, vs. control ^b^			<0.0001 **		>0.99		<0.0001 **		
*p* value, vs. PG ^b^							<0.0001 **		

^a^ *p* values are calculated using the unpaired *t*-test or Fisher’s exact test. ^b^ *p* values are calculated using Tukey’s HSD test or Fisher’s exact test between each pair of groups. ** *p* < 0.01, * *p* < 0.05. PG, primary open-angle glaucoma; EG, exfoliation glaucoma; BMI, body mass index; sBP, systolic blood pressure; dBP, diastolic blood pressure; bpm, beats per minute; DM, diabetes mellitus; PEM, pseudoexfoliation material; SD, standard deviation; CI, confidence interval.

**Table 2:** Comparison of HRV between control group and glaucoma (+) group, and between control group and PG group and EG group.

Parameters	Control		Glaucoma		PG		EG		*p* Value ^a^
	Mean ± SD	95% CI	Mean ± SD	95% CI	Mean ± SD	95% CI	Mean ± SD	95% CI	
LnTP	6.14 ± 0.98	5.94, 6.33	6.19 ± 0.90	6.12, 6.25	6.24 ± 0.89	6.16, 6.32	6.04 ± 0.90	5.91, 6.17	0.03 *
*p* value, vs. control ^b^			0.61		0.56		0.69		
*p* value, vs. PG ^b^							0.03 *		
LnVLF	5.56 ± 0.80	5.40, 5.72	5.61 ± 0.74	5.55, 5.66	5.65 ± 0.75	5.59, 5.72	5.48 ± 0.69	5.38, 5.58	0.02 *
*p* value, vs. control ^b^			0.59		0.52		0.65		
*p* value, vs. PG ^b^							0.02 *		
LnLF	4.28 ± 1.44	3.99, 4.57	4.06 ± 1.41	3.95, 4.16	4.16 ± 1.35	4.05, 4.28	3.78 ± 1.52	3.56, 3.99	0.002 *
*p* value, vs. control ^b^			0.15		0.74		0.01 *		
*p* value, vs. PG ^b^							0.04 *		
LnHF	4.21 ± 1.52	3.90, 4.52	4.46 ± 1.40	4.35, 4.56	4.53 ± 1.36	4.41, 4.65	4.26 ± 1.47	4.05, 4.47	0.02 *
*p* value, vs. control ^b^			0.11		0.10		0.96		
*p* value, vs. PG ^b^							0.06		
LnLF/LnHF	20.22 ± 4.37	19.30, 21.07	20.30 ± 3.98	20.01, 20.60	20.58 ± 3.90	20.24, 20.91	19.56 ± 4.12	18.97, 20.15	0.01 *
*p* value, vs. control ^b^			0.79		0.65		0.42		
*p* value, vs. PG ^b^							0.008 *		

^a^ *p* values are calculated using the unpaired *t*-test. ^b^ *p* values are calculated using Tukey’s HSD test. * *p* < 0.05. HRV, heart rate variability; PG, primary open-angle glaucoma; EG, exfoliation glaucoma; SD, standard deviation; CI, confidence interval; LnTP, natural logarithm Total Power; LnVLF, natural logarithm Very Low Frequency Power; LnLF, natural logarithm Low Frequency; LnHF, natural logarithm High Frequency.

**Table 3:** Multivariate analysis for possible parameters associated with LnTP.

Parameters	Estimate	95% CI	*p* Value
Age, /year	−0.01	−0.02, −0.01	<0.0001 **
Sex, F/M	0.02	−0.06, 0.06	0.99
Smoking habit, yes/no	0.16	−0.18, 0.20	0.93
BMI, /kg/m^2^	−0.04	−0.04, −0.00	0.04 *
sBP, /mmHg	0.00	−0.00, 0.01	0.18
dBP, /mmHg	−0.00	−0.007, 0.01	0.87
Pulse rate, /bpm	−0.03	−0.03, −0.02	<0.0001 **
Hypertension, yes/no	−0.13	−0.14, −0.01	0.03 *
DM, yes/no	−0.07	−0.14, 0.03	0.21
PEM deposition, yes/no	0.00	−0.09, 0.06	0.73
Glaucoma, yes/no	0.10	−0.08, 0.11	0.78

*p* values are calculated using the generalized regression model. ** *p* < 0.01, * *p* < 0.05. BMI, body mass index; sBP, systolic blood pressure; dBP, diastolic blood pressure; bpm, beats per minute; DM, diabetes mellitus; PEM, pseudoexfoliation material; CI, confidence interval.

**Table 4:** Multivariate analysis for possible parameters associated with LnVLF.

Parameters	Estimate	95% CI	*p* Value
Age, /year	−0.01	−0.02, −0.01	<0.0001 **
Sex, F/M	−0.02	−0.07, 00	0.42
Smoking habit, yes/no	0.02	−0.13, 0.18	0.78
BMI, /kg/m^2^	−0.02	−0.03, 0.00	0.056
sBP, /mmHg	0.00	−0.00, 0.01	0.14
dBP, /mmHg	−0.00	−0.01, 0.00	0.72
Pulse rate, /bpm	−0.02	−0.03, −0.02	<0.0001 **
Hypertension, yes/no	−0.05	−0.10, 0.01	0.084
DM, yes/no	−0.04	−0.11, 0.04	0.33
PEM deposition, yes/no	−0.02	−0.08, 0.05	0.62
Glaucoma, yes/no	0.02	−0.06, 0.09	0.70

*p* values are calculated using the generalized regression model. ** *p* < 0.01. BMI, body mass index; sBP, systolic blood pressure; dBP, diastolic blood pressure; bpm, beats per minute; DM, diabetes mellitus; PEM, pseudoexfoliation material; CI, confidence interval.

**Table 5:** Multivariate analysis for possible parameters associated with LnLF.

Parameters	Estimate	95% CI	*p* Value
Age, /year	−0.03	−0.03, −0.02	<0.0001 **
Sex, F/M	0.01	−0.09, 0.11	0.87
Smoking habit, yes/no	−0.12	−0.42, 0.19	0.45
BMI, /kg/m^2^	−0.03	−0.06, −0.00	0.04 *
sBP, /mmHg	0.00	−0.00, 0.01	0.47
dBP, /mmHg	0.01	−0.00, 0.02	0.21
Pulse rate, /bpm	−0.02	−0.03, −0.02	<0.0001 **
Hypertension, yes/no	−0.15	−0.26, −0.05	0.004 *
DM, yes/no	−0.12	−0.25, 0.02	0.10
PEM deposition, yes/no	−0.04	−0.16, 0.08	0.52
Glaucoma, yes/no	−0.05	−0.21, 0.10	0.51

*p* values are calculated using the generalized regression model. ** *p* < 0.01, * *p* < 0.05. BMI, body mass index; sBP, systolic blood pressure; dBP, diastolic blood pressure; bpm, beats per minute; DM, diabetes mellitus; PEM, pseudoexfoliation material; CI, confidence interval.

**Table 6:** Multivariate analysis for possible parameters associated with LnHF.

Parameters	Estimate	95% CI	*p* Value
Age, /year	−0.01	−0.02, −0.00	0.02 *
Sex, F/M	0.02	−0.08, 0.12	0.73
Smoking habit, yes/no	0.16	−0.14, 0.47	0.29
BMI, /kg/m^2^	−0.04	−0.07, −0.01	0.01 *
sBP, /mmHg	0.00	−0.00, 0.01	0.56
dBP, /mmHg	−0.00	−0.01, 0.01	0.88
Pulse rate, /bpm	−0.03	−0.04, −0.03	<0.0001 **
Hypertension, yes/no	−0.13	−0.23, −0.02	0.02 *
DM, yes/no	−0.07	−0.21, 0.07	0.33
PEM deposition, yes/no	−0.04	−0.16, 0.08	0.50
Glaucoma, yes/no	0.10	−0.06, 0.25	0.22

*p* values are calculated using the generalized regression model. ** *p* < 0.01, * *p* < 0.05. BMI, body mass index; sBP, systolic blood pressure; dBP, diastolic blood pressure; bpm, beats per minute; DM, diabetes mellitus; PEM, pseudoexfoliation material; CI, confidence interval.

**Table 7:** Multivariate analysis for possible parameters associated with LF/HF.

Parameters	Estimate	95% CI	*p* Value
Age, /year	−0.06	−0.08,−0.04	<0.0001 **
Sex, F/M	0.00	−0.28, 0.28	0.99
Smoking habit, yes/no	0.08	−0.77, 0.92	0.86
BMI, /kg/m^2^	−0.10	−0.18, −0.02	0.01 *
sBP, /mmHg	0.01	−0.01, 0.03	0.29
dBP, /mmHg	0.00	−0.02, 0.03	0.78
Pulse rate, /bpm	−0.11	−0.13, −0.09	<0.0001 **
Hypertension, yes/no	−0.40	−0.69, −0.11	0.007 *
DM, yes/no	−0.28	−0.66, 0.11	0.16
PEM deposition, yes/no	−0.11	−0.44, 0.22	0.52
Glaucoma, yes/no	0.07	−0.36, 0.50	0.74

*p* values are calculated using the generalized regression model. ** *p* < 0.01, * *p* < 0.05. BMI, body mass index; sBP, systolic blood pressure; dBP, diastolic blood pressure; bpm, beats per minute; DM, diabetes mellitus; PEM, pseudoexfoliation material; CI, confidence interval.

## Data Availability

The raw data supporting the conclusions of this article will be made available by the authors on request.

## Abbreviations

The following abbreviations are used in this manuscript:

HRV	Heart rate variability
ANS	Autonomic nervous system
SDNN	The standard deviation of normal-to-normal intervals
RMSSD	The square root of the mean of the sum of the squared differences between adjacent normal-to-normal intervals
CVRR	The coefficient of variation of R-R intervals
PG	Primary open-angle glaucoma
EG	Exfoliation glaucoma
NTG	Normal tension glaucoma
PEM	Pseudoexfoliation material
IOP	Intraocular pressure
BMI	Body mass index
BP	Blood pressure
CI	Confidence interval
XFS	Exfoliation syndrome
TP	Total power
VLF	Very-low-frequency
LF	Low-frequency
HF	High-frequency
